# Radioulnar Instability and Ulnar Stump Stabilization in Distal Radio Ulnar Joint Arthritis: A Cadaver Study

**DOI:** 10.7759/cureus.41163

**Published:** 2023-06-29

**Authors:** Takeru Ohara, Takahiro Yamazaki, Yusuke Matsuura, Takane Suzuki, Seiji Ohtori

**Affiliations:** 1 Orthopedic Surgery, Chiba University's Graduate School of Medicine, Chiba, JPN; 2 Orthopaedics, Chiba University Hospital, Chiba, JPN

**Keywords:** druj salvage procedures, extensor tendon, druj, darrach procedure, sauvé-kapandji procedure

## Abstract

Background: Salvage procedures for distal radioulnar joint (DRUJ) arthritis, like the Darrach or Sauvé-Kapandji (S-K) procedures, often result in extensor tendon ruptures at the ulnar stump. Radioulnar instability is considered the underlying cause and stump stabilization techniques are employed. This study investigated radioulnar instability, extensor tendon irritation, and the effectiveness of stump stabilization techniques following salvage procedures.

Methods: Six upper limbs from three cadavers were used. Forearm rotation was measured using magnetic position sensors to assess radial movement. The Darrach procedure was performed on two limbs, comparing radial motion ranges for different ulnar osteotomy positions. The risk of tendon rupture was assessed with applied weight. The S-K procedure was performed on four limbs, evaluating stump stabilization techniques and radial movement distance underweight.

Results: Proximal osteotomy positions increased radial motion range. Extensor tendon irritation occurred when the load was applied to the volar and ulnar sides, particularly with a pronated forearm. Stump stabilization techniques did not significantly contribute to ulnar stump stabilization.

Conclusions: Proximal ulnar osteotomy positions in DRUJ salvage procedures led to increased radioulnar instability and potential complications. Load application on the volar and ulnar sides, especially in a pronated forearm, increased the risk of tendon rupture. Stump stabilization techniques showed limited utility in stabilizing the ulnar stump or reducing complications. These findings can inform strategies for minimizing complications in DRUJ salvage procedures.

## Introduction

Patients suffering from distal radioulnar joint (DRUJ) disorders related to osteoarthritis and rheumatoid arthritis commonly experience ulnar side wrist pain and subcutaneous extensor tendon rupture [1]. Salvage procedures like the Darrach or Sauvé-Kapandji (S-K) procedure have shown positive outcomes for patients who do not respond well to conservative treatment [2-4]. However, complications related to the stump, such as pain and instability at the resected proximal ulnar stump and subcutaneous extensor tendon rupture, have been reported following the Darrach and S-K procedures [5-7].

Subcutaneous extensor tendon rupture can be caused by radioulnar instability and the absence of soft tissue interposition between the tendons and the ulnar stump [6]. Previous studies have indicated that the ulnar osteotomy should be performed as distally as possible to preserve the interosseous membrane, contributing to radioulnar stability [8]. Millroy et al. observed that clicking of the stump was more likely to occur when the stump was positioned 25 mm or more away from the joint surface after the S-K procedure [9]. Conversely, Wada et al. reported that distal osteotomy positions could result in impingement of the extensor tendon between the proximal stump of the ulna and the extensor retinaculum, suggesting that the stump should be positioned at least 25 mm proximal to the joint surface [7].

Previous studies, such as those conducted by Douglas et al., who examined radioulnar instability after hemi-resection of the ulnar head, Darrach procedure, and DRUJ arthroplasty [10], and Kitamura et al., who assessed DRUJ stability using fresh frozen cadavers [11], have measured the movement distance between the radius and ulna after applying a load. However, no study has investigated the movement between the radius and ulna based on the osteotomy site of the ulna or the effects of the stump on the extensor tendons during forearm rotation.

Tenodesis stabilization has been proposed to improve stump instability. Minami et al., Chu et al., and Leslie et al. reported favorable outcomes using stump stabilization with the extensor carpi ulnaris (ECU) tendon [12-14], while Breen et al. successfully employed the ECU and flexor carpi ulnaris (FCU) tendons [15]. On the other hand, Sauerbier et al. found that stabilization using the pronator quadratus muscle (PQ) or the ECU and FCU tendons did not yield significant results in their study using fresh frozen cadavers [16], indicating uncertainty regarding the effectiveness of stabilization.

Based on our hypothesis that radioulnar instability following the Darrach or S-K procedure is influenced by the position of the ulnar osteotomy, postoperative limb position, and applied load and that stabilization procedures can improve stability, this study aimed to evaluate the instability of the ulnar stump and extensor irritation after the Darrach procedure considering the osteotomy site and forearm rotation, as well as assess the impact of stabilization techniques.

## Materials and methods

Specimens

Ethical approval was obtained from the Institutional Review Board of Chiba University's Graduate School of Medicine, Chiba, Japan, to conduct this study with approval number- 3224. Upper limbs from fresh frozen cadavers donated to the clinical anatomical laboratory were utilized (six upper limbs from three cadavers). The right and left upper limbs of an 84-year-old woman was used for investigating radioulnar instability and extensor tendon irritation (Experiments 1 and 2). To assess the effectiveness of stump stabilization procedures (Experiment 3), four upper limbs from two men (aged 103 and 99) were employed. After removing the dorsal skin, the extensor tendons were dissected while preserving the extensor retinaculum.

Jig and fixation

**Figure 1:**
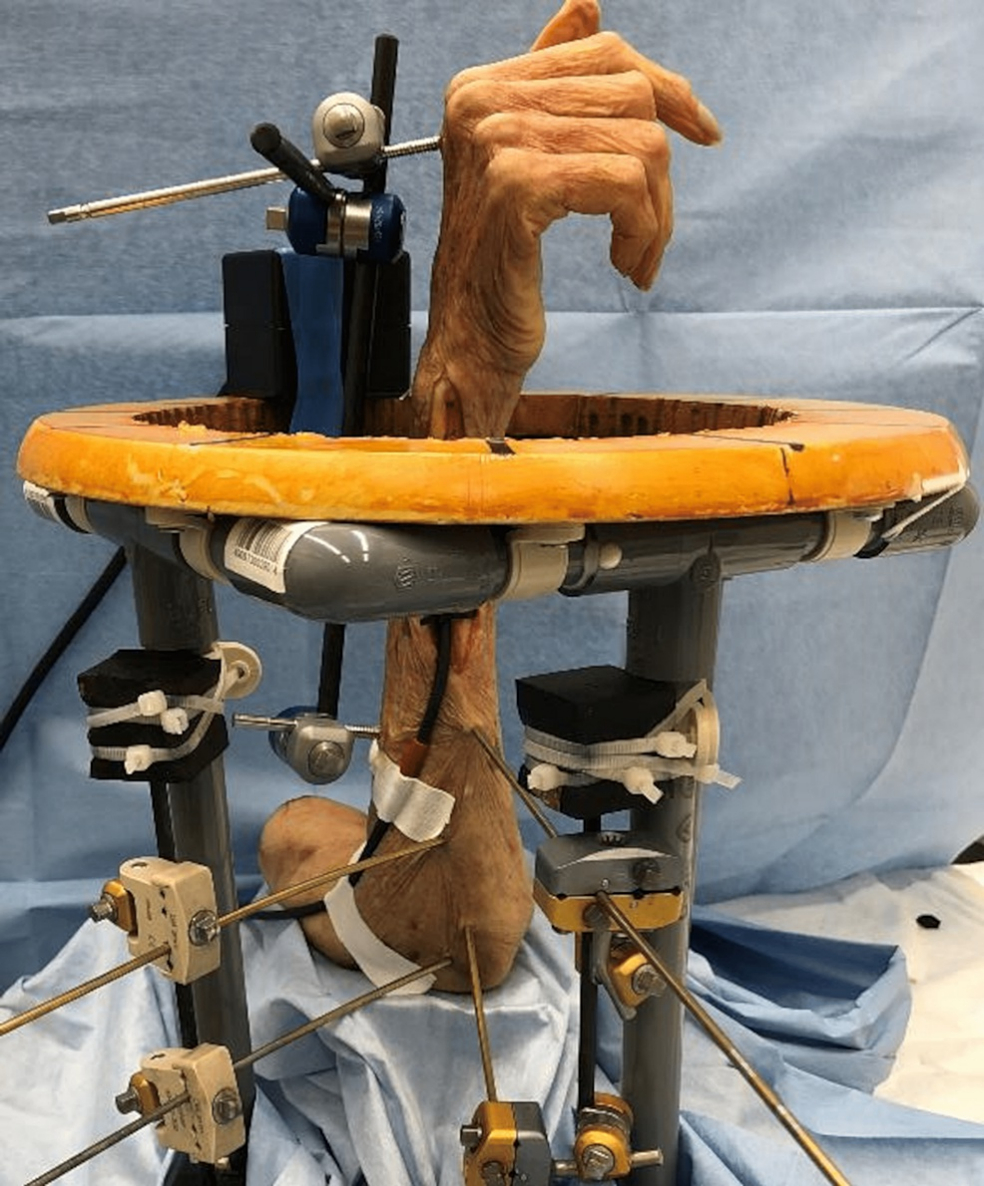
An original jig with a cylinder was used, the ulna was fixed with a K-wire, and the wrist was fixed using an external fixator in the neutral position

An original jig consisting of a cylinder was utilized for the experiment. The ulna was fixed to the jig with K-wires, ensuring that the elbow flexion was set at 90 degrees and the fovea of the ulna, serving as the axis of forearm rotation, was positioned at the center of the circle. The wrist was immobilized using an external fixator (Hoffmann II, Stryker, Kalamazoo, Michigan) in a neutral position (Fig 1).

Biomechanical evaluation 

**Figure 2:**
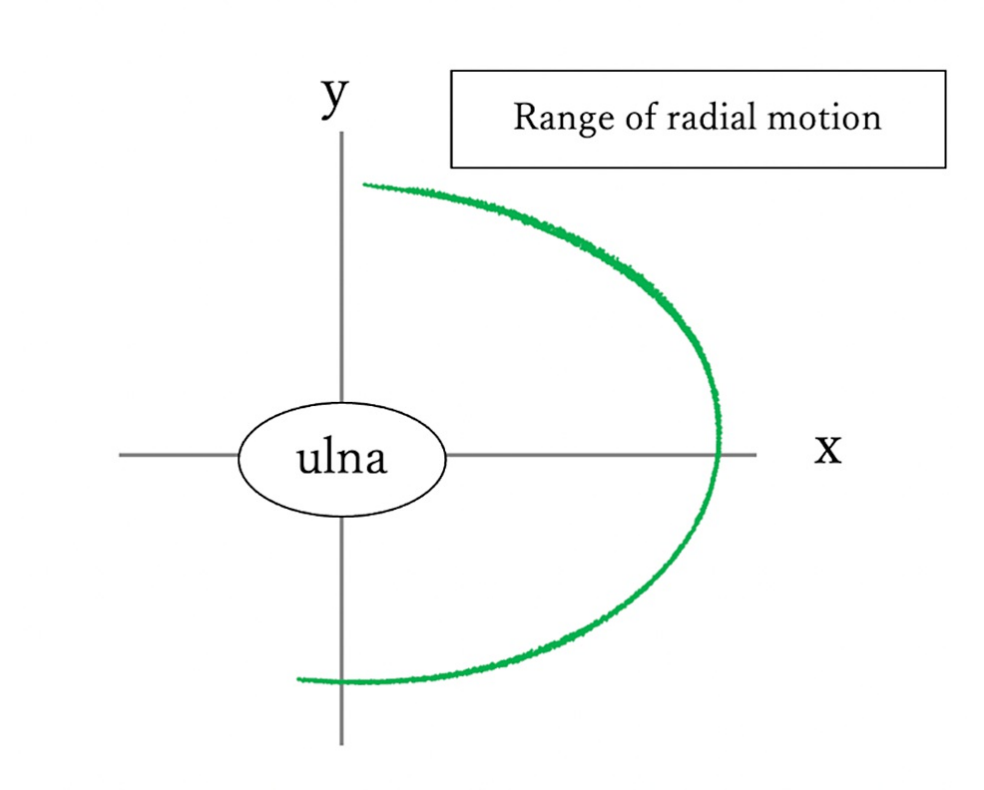
The range of radial motion centered on the ulna

A magnetic position sensor (Fastrak, Polhemus, Inc., Colchester, VT) was employed for the study. Sensors were placed on the radial styloid process and the ulnar shaft while the transmitter was fixed onto the jig. The coordinates of the ulna were adjusted by measuring the distance from the ulna sensor to the center of rotation, thereby plotting the position of the radius in XY coordinates, depicting the movement of the radius around the ulna. The coordinates of the radius were measured using a sensor through manual pronation and supination, repeated approximately 10 times to record and plot approximately 10,000 coordinates on a coordinate plate, enabling the illustration of the movement of the semicircular radius centered on the ulna (Fig 2).

Experiment 1: Range of Radial Motion and Ulnar Osteotomy Position 

**Figure 3:**
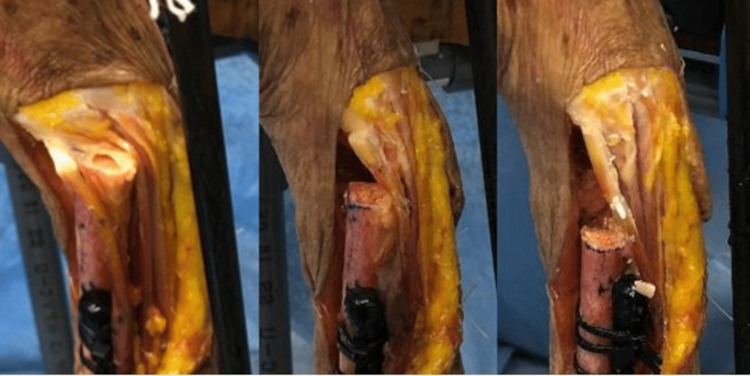
Osteotomy position at 10, 25, and 40 mm from the joint surface

We examined how the position of the ulnar osteotomy influences the range of radial motion. Following the osteotomy and excision of the ulna head, a passive movement was used to measure the range of radial motion, observing any changes at the osteotomy site. The osteotomy positions were set at 10, 25, and 40 mm from the joint surface (Fig 3), and the range of radial motion was illustrated and compared.

Experiment 2: Risk of Extensor Tendon Rupture 

**Figure 4:**
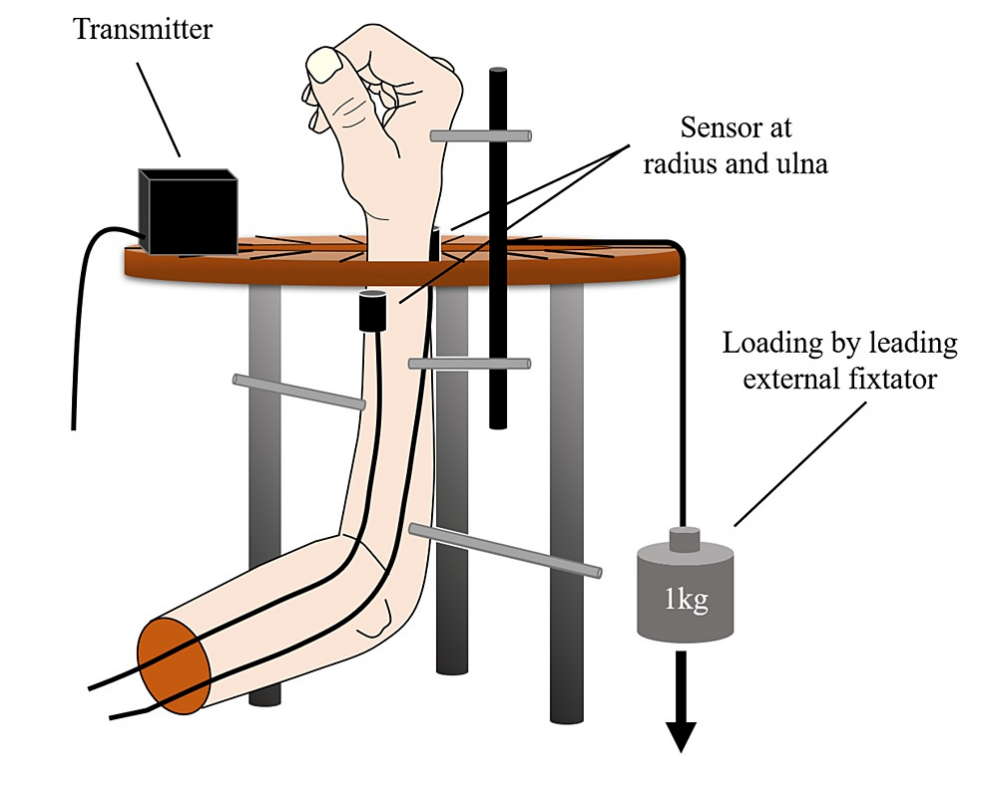
A load was applied using a weight of 1 kg

We investigated the risk of extensor tendon rupture caused by stump irritation. "Irritation" was defined as the contact between the tendon and stump. The area where the stump came into contact with the extensor digitorum communis (EDC) tendon, known to be susceptible to tendon rupture from repetitive contact, was defined as the irritation area. Conversely, the non-irritation area was defined as the region where the stump did not make contact, determined by gross observation. These areas were predetermined through manual manipulation of the radius. The forearm was positioned in neutral, pronated at 30° and 60°, and supinated at 30° and 60°. A load of 1 kg was applied to the dorsal, volar, radial, and ulnar sides using an external fixator based on a previous study (Fig 4) [10]. The position of the radius was recorded, and the risk of extensor tendon rupture due to forearm position and the applied load was evaluated based on the coordinates falling within the irritation or non-irritation areas. This examination was performed at 10, 25, and 40 mm osteotomy positions.

Experiment 3: Evaluation of Stump Stabilization Techniques 

We evaluated the effectiveness of various stump stabilization techniques. The S-K procedure was performed following previous reports [17], with the radius and ulnar head fixed using K-wires. The stump was positioned 25 mm from the joint surface. To assess instability, a 1 kg load was applied to the dorsal, volar, radial, and ulnar sides in the neutral position, pronated at 30° and 60°, and supinated at 30° and 60°, as in Experiment 2. The movement distance was measured by tracking changes in the coordinates of the radius. The stump stabilization techniques used were as follows.

Stabilization with PQ suturing: Following the approach described by Johnson et al., the PQ attachment portion was separated from the ulna and used to cover the stump after osteotomy [18]. It was then sutured to the ECU fascia.

**Figure 5:**
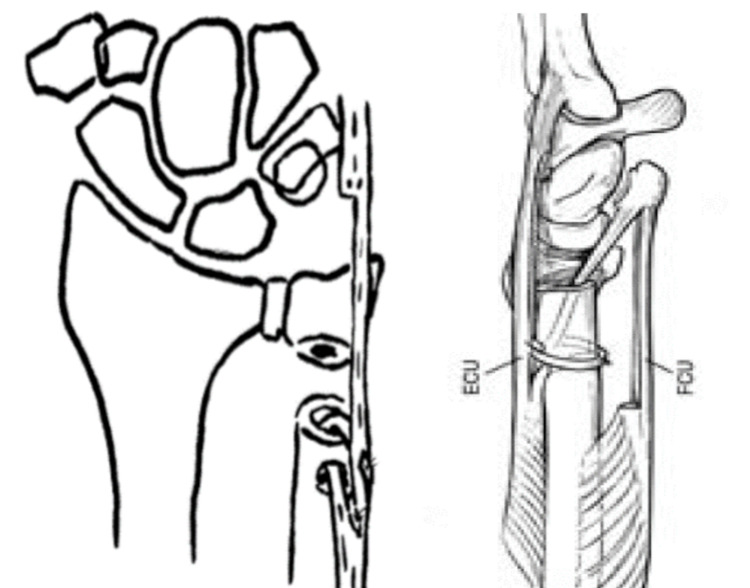
Stabilization by ECU, stabilization by ECU and FCU ECU:Extensor Carpi Ulnaris, FCU:Flexor Carpi Ulnaris

Stabilization with ECU half-slip tendon: In accordance with the method outlined by Minami et al., a 3.5-mm hole was drilled from the dorsoulnar aspect of the ulnar shaft into the intramedullary cavity [12]. The ECU tendon was split at the central sulcus, with the radial half released at the ulnocarpal level. The proximally based strip was rotated and left attached at the musculotendinous junction. Subsequently, this strip was passed into the medullary canal through the drilled hole, retrieved at the distal stump of the ulna, and then sutured back onto itself in an interlacing manner. The ECU tendon sheath was then sutured (Fig 5).

Stabilization with ECU and FCU half-slip tendon: Based on the technique described by Breen et al., a distally-based tendon strip of the FCU and a proximally-based tendon strip of the ECU were created [16]. The FCU strip was passed proximally through the open medullary canal of the osteotomized ulna and exited through a vertically oriented drill hole in the ulnar diaphysis. The ECU strip was then threaded through the same drill hole, interwoven with the FCU strip, and secured with a suture (Fig 5).

Stabilization with FCU half-slip tendon: This procedure was identical to procedure #3, except that only the FCU half-slip tendon was used to stabilize the stump.

**Figure 6:**
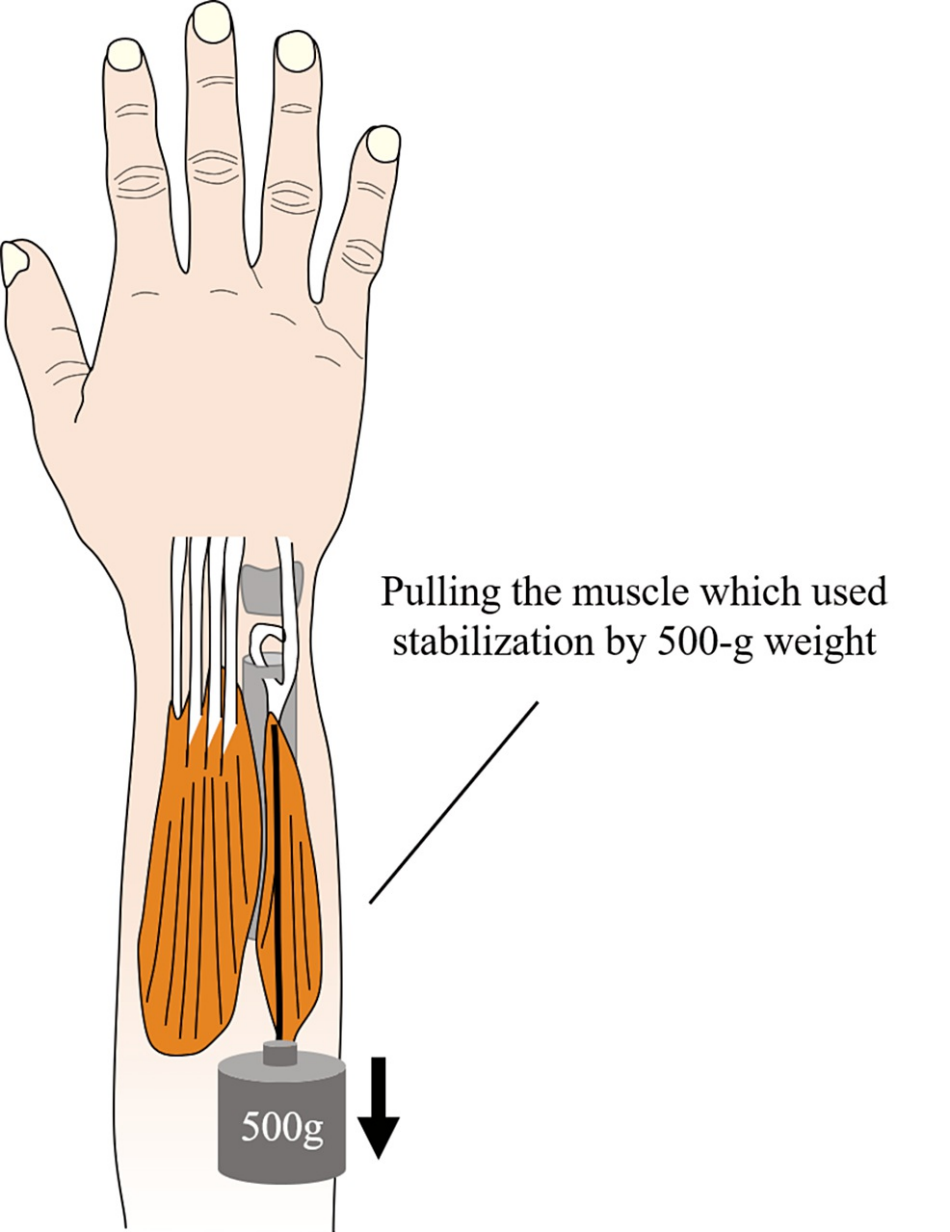
The muscles and tendons which used for stabilization were pulled using a 500-g weight

For the ECU or FCU stabilization procedures, the muscles and tendons employed for stabilization were tensioned using a 500-g weight (Fig 6). A 1-kg load was applied to each group, including the no stabilization group, PQ stabilization group, ECU stabilization group, ECU and FCU stabilization group, and FCU stabilization group, using the same method as Experiment 2. The movement distance was calculated based on the change in coordinates of the radius, and the movement distances of the groups were compared.

Data analysis

To account for variations among specimens, the movement in the no-stabilization group was standardized to 1, and the movement in the other groups was expressed as a ratio relative to the no-stabilization group. The movement in each group was compared for the four specimens. Statistical analysis was performed using Wilcoxon T-tests, with a significance level of p < 0.05.

## Results

Experiments 1 and 2

**Figure 7:**
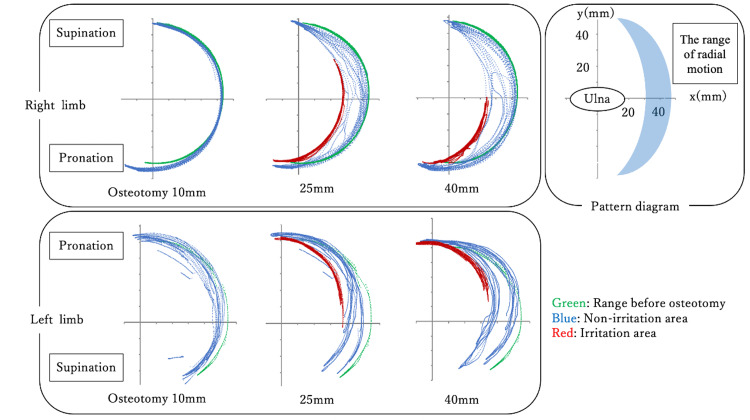
The range of radial motion according to the osteotomy position upper: right limb, lower: left limb, upper right: pattern diagram; green indicates range of movement before osteotomy, blue indicates the non-irritation area, and red indicates the irritation area.

The range of radial motion is illustrated for each ulnar osteotomy position (Fig 7). The upper row represents the right limb, the lower row represents the left limb, and the pattern diagram is displayed in the upper right corner. The color green represents the range of motion before osteotomy, blue represents the non-irritation area where the stump does not contact the EDC tendon, and red indicates the irritated area where the stump comes into contact with the EDC tendon. The range of radial motion increased as the osteotomy position moved proximally to 10 mm, 25 mm, and 40 mm from the joint surface. At the 10 mm osteotomy position, there were no areas where the stump was likely to irritate the EDC tendon, and impingement of the extensor tendon between the proximal stump of the ulna and the extensor retinaculum did not occur. However, irritation areas were observed at the 25 mm and 40 mm osteotomy positions. At the 40 mm osteotomy position, the irritation area was limited to the pronation position. Both upper limbs exhibited similar findings.

The results of the loading tests are presented in Table 1. In the right limb, when the ulnar osteotomy was performed at 25 mm, the stump was in the irritation area when the limb was loaded on the volar or ulnar side with a pronation angle of 60° or loaded on the ulnar side with a pronation angle of 30°. Otherwise, the stump remained in the non-irritation area. If the ulnar osteotomy was performed at 40 mm, the stump was in the irritation area when the limb was loaded on the volar or ulnar side with pronation angles of 30° or 60° or loaded on the ulnar side with the limb in a neutral position. In the left limb, when the osteotomy was performed at 25 mm, the stump was in the irritation area when the limb was loaded on the volar and ulnar sides with pronation angles of 30° and 60°. Similar findings were observed when the osteotomy was performed at 40 mm, and when the limb was loaded on the volar side in a neutral position, the stump was in the irritation area. In both upper limbs, the stump was consistently in the irritation area when the limb was loaded on the volar and ulnar sides in a pronated position.

Experiment 3

**Figure 8:**
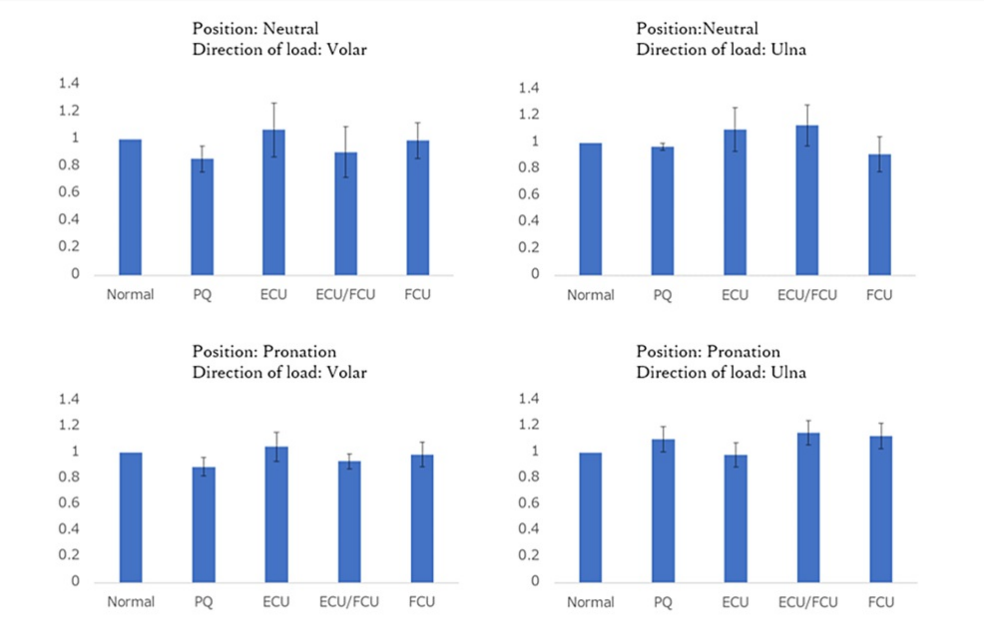
The radius movement distance in the no stabilization group was set as 1, and the average moving distances when stabilizing procedures were applied to the stump were expressed as a ratio. Each bar represents the mean value with standard error. PQ: Pronator Quadratus; ECU: Extensor Carpi Ulnaris; FCU: Flexor Carpi Ulnaris

Based on the findings of Experiment 2, which indicated a risk of extensor tendon rupture when loads were applied to the upper limb on the volar and ulnar sides in neutral and pronation positions, the effectiveness of the stabilization technique was examined in those scenarios. Figure 8 presents the results, where the movement distance of the stump in the no stabilization group was set as the reference value of 1, and the movement distances when the stump was stabilized using different techniques were expressed as ratios. Each bar represents the mean value with standard error. None of the techniques demonstrated a statistically significant reduction in the movement distance of the stump.

## Discussion

This study observed that the range of motion between the radius and ulna increased as the ulnar osteotomy was performed further from the articular plane. At osteotomy positions of 25 mm and 40 mm, there was an area of irritation where the stump came into contact with the extensor tendon. This suggests that as the ulnar osteotomy position becomes more proximal, the increased range of motion may elevate the risk of stump-related pain, instability, and extensor tendon rupture. Unlike a previous study [7], we did not observe impingement of the extensor tendon between the proximal stump of the ulna and the extensor retinaculum with a 10 mm osteotomy. Consistent with findings from previous research, it is therefore advisable to position the stump as distally as possible [8].

However, suppose there was initially a dorsal dislocation of the ulnar head in DRUJ. In that case, the stump should be positioned so that it does not protrude dorsally and does not irritate the radius. During the performance of the S-K procedure, creating a gap between the ulnar head and the stump is necessary to prevent fusion of the distal and proximal ulnar ends.

Furthermore, we observed that the stump moved into a position that contacted the EDC tendon when a load was applied to the volar or ulnar side with the forearm in a pronated position. This corresponds to daily activities such as holding an object with the forearm horizontally in a neutral or pronated position. Our findings suggest that even holding a 1 kg load in such forearm positions could lead to tendon irritation. Therefore, postoperative rehabilitation and guidance in daily activities should be considered, including instructing patients to hold objects in a supinated forearm position whenever possible.

We found that stump stabilization did not significantly improve radioulnar instability. Previous clinical studies have reported the usefulness of various stabilization techniques [12-15], but no direct comparisons have been made. Another study using fresh frozen cadavers found no benefit from stabilization techniques. These techniques may not contribute to stabilization or reduce pain or clicking. However, covering the stump itself may reduce the risk of tendon rupture.

In conclusion, when performing the Darrach or S-K procedure, it is advisable to position the stump distally, considering the risk of extensor tendon rupture and other factors, and there may be no need for a stump stabilization procedure. However, in clinical practice, the stump position during the S-K procedure often ends up approximately 20-25 mm from the joint surface due to the creation of the ulnar gap, making it challenging to position it more distally and avoid an irritation area that poses a risk of extensor tendon rupture. To minimize direct stimulation of the stump measures such as bluntly scraping the bone may be considered [19].

The first limitation of this study is the small sample size. However, experiments 1 and 2 yielded similar results for both limbs, indicating that the range of motion between the radius and ulna and the irritation of the stump on the extensor tendon are influenced by the osteotomy and forearm position. The second limitation is that the assessment of forearm muscles was conducted in a non-contracted state. Several previous studies have also evaluated the instability of salvage procedures in fresh frozen cadavers [10-11], and Douglas et al. noted that while these results may be exaggerated due to the absence of muscle contraction, they are still less variable and more comparable [10]. Third, the cadavers used in this study did not have DRUJ deformations. Some patients, particularly those with rheumatoid arthritis, may experience instability, so this study may not fully reflect actual clinical practice.

Nevertheless, we believe it was still possible to assess the effects of stump position, load direction, and the usefulness of stabilization techniques. Fourth, the cadaver specimens, being elderly, may have exhibited degeneration of the interosseous membrane, triangular fibrocartilage complex, and extensor tendons. Fifth, the measurement of the range of radial motion relied on passive movement, which could introduce variation in the observed range.

## Conclusions

In conclusion, this study utilized fresh frozen cadavers to investigate radioulnar instability following salvage procedures for DRUJ arthritis, the risk of extensor tendon rupture at the stump, and the effectiveness of ulnar stump stabilization techniques. The results suggest that the range of motion between the radius and ulna increases as the ulnar osteotomy position becomes more proximal, heightening the risk of complications. Furthermore, when the stump is loaded on the volar or ulnar side during pronation, it irritates the extensor tendon, increasing the risk of tendon rupture. This study did not observe the previously reported benefits of stump stabilization techniques. We propose that when performing the Darrah or S-K procedure on the distal radioulnar joint, reducing stump instability and associated complications can be achieved by positioning the ulnar osteotomy as distally as possible and by avoiding pronated forearm positions, particularly on the volar and ulnar sides.
